# Supplementary material for: Co-isolation of genetically distinct Burkholderia pseudomallei strains from a single patient in North Queensland
Source: PLoS One. 2025 Dec 18;20(12):e0338333. doi: 10.1371/journal.pone.0338333 (PMC12714287; doi:10.1371/journal.pone.0338333)
Supplement: S1 Fig — (PDF) [file pone.0338333.s001.pdf]

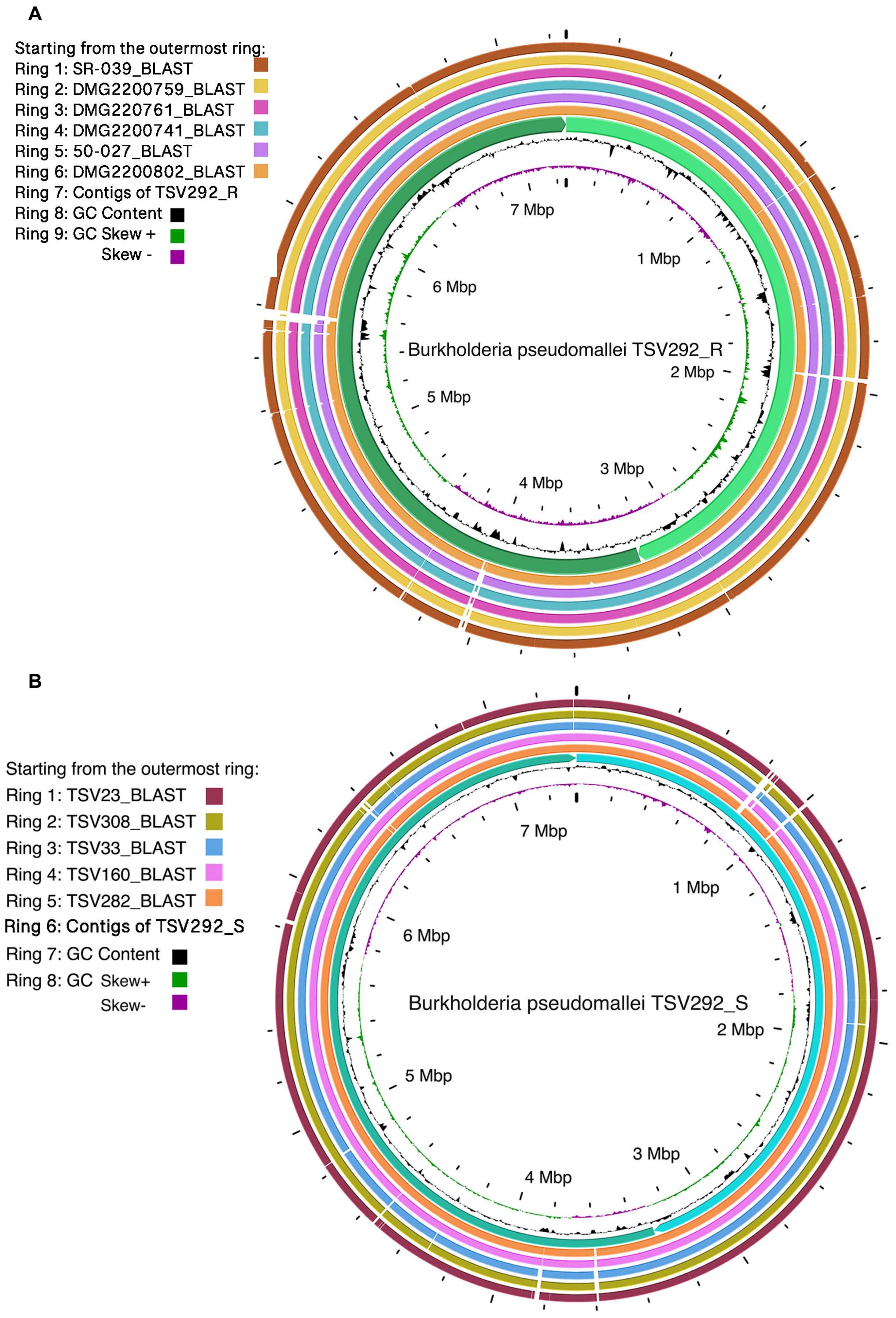

S1 Fig. Genome alignment of TSV292_1 (rough) and TSV292_2 (smooth) with their respective closest isolates genomes.
