# Supplementary material for: Co-isolation of genetically distinct Burkholderia pseudomallei strains from a single patient in North Queensland
Source: PLoS One. 2025 Dec 18;20(12):e0338333. doi: 10.1371/journal.pone.0338333 (PMC12714287; doi:10.1371/journal.pone.0338333)
Supplement: S2 Fig — MAUVE alignment of both TSV292_1 (rough) and TSV292_2 (smooth) against each other. The red line delimits both chromosomes in each genome. Contig 1 is one the left and contig 2 is on the right. Each color corresponds to an identical region in both genomes. (PDF) [file pone.0338333.s002.pdf]

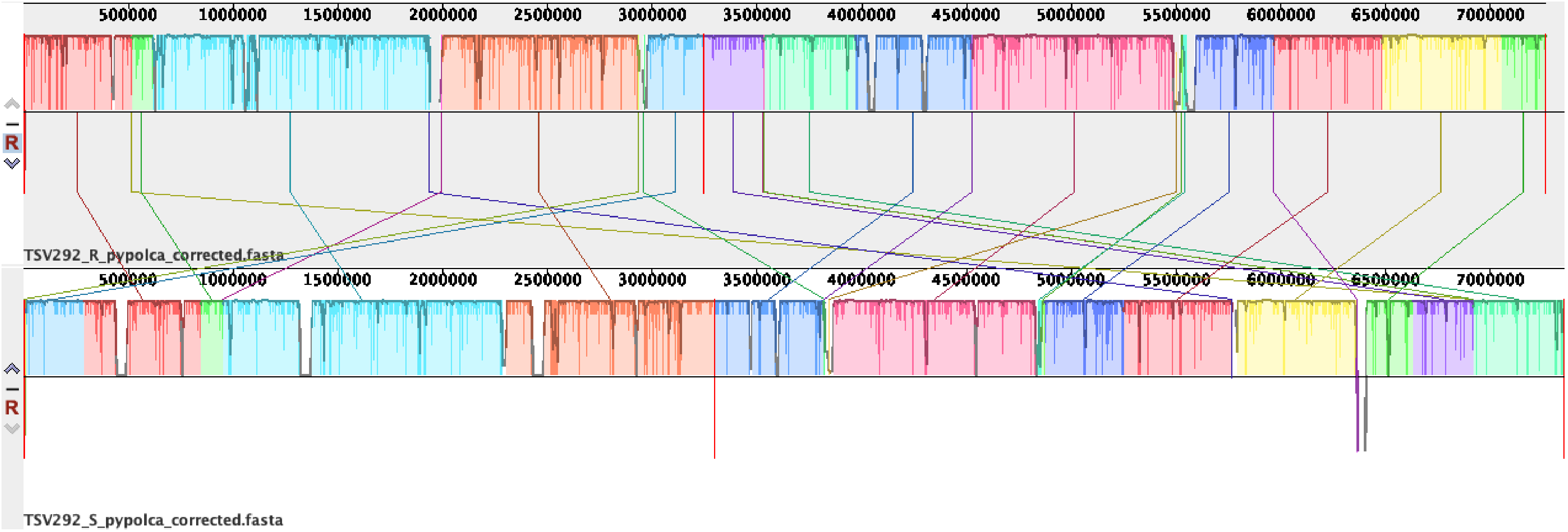

S2 Fig. MAUVE alignment of both TSV292_1 (rough) and TSV292_2 (smooth) against each other. The red line delimits both chromosomes in each genome. Contig 1 is one the left and contig 2 is on the right. Each color correspond to an identical region in both genome.
