# Supplementary material for: Co-isolation of genetically distinct Burkholderia pseudomallei strains from a single patient in North Queensland
Source: PLoS One. 2025 Dec 18;20(12):e0338333. doi: 10.1371/journal.pone.0338333 (PMC12714287; doi:10.1371/journal.pone.0338333)
Supplement: S3 Fig — Genome alignment of TSV292_2 (smooth) on TSV292_1 (rough) using LASTZ alignment tool. Forward alignments between the reference TSV292_1 (rough) contig 1 (A) and contig 2 (B) (x-axis) and TSV292_2 (smooth) query (y-axis) genomes are shown in blue, indicating regions of conserved synteny in the same orientation. Alignments in red represent sequences aligned in the reverse-complement orientation, corresponding to inverted segments relative to the reference. Gaps with no alignment indicate regions unique to one genome, missing from the other, or below the similarity threshold for alignment. (PDF) [file pone.0338333.s003.pdf]

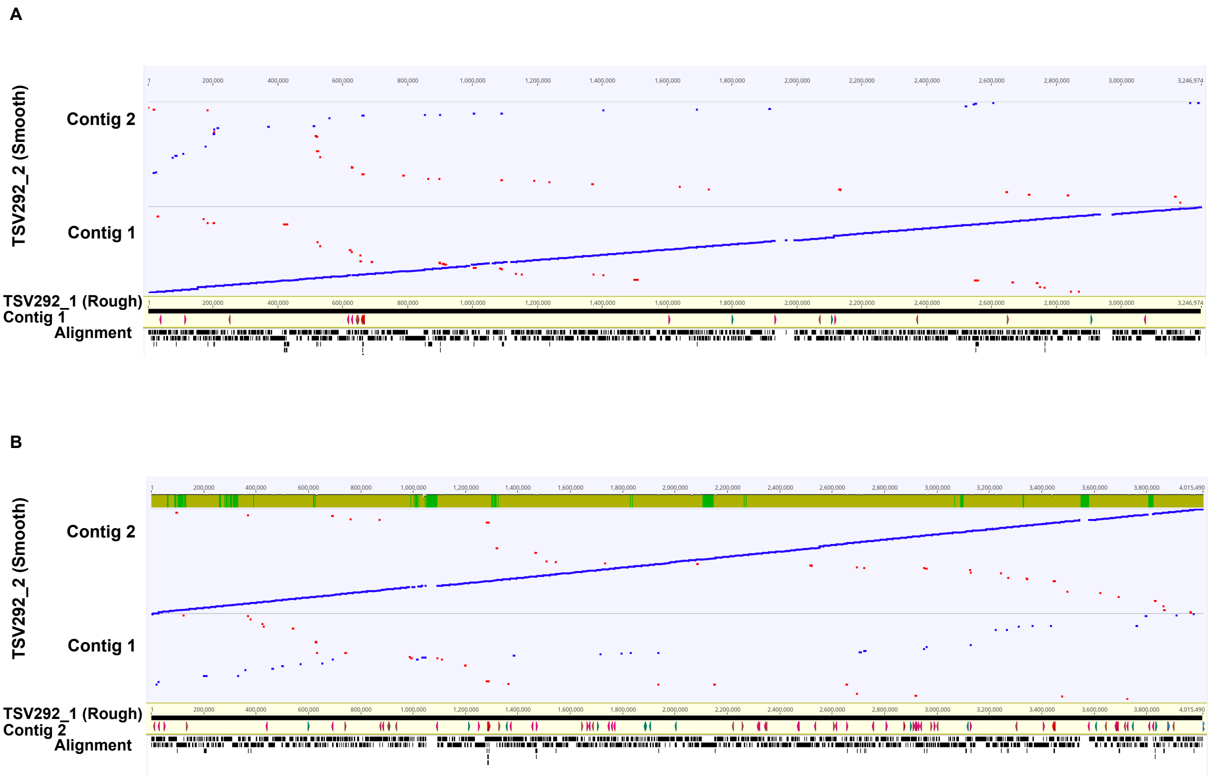

S3 Fig. Genome alignment of TSV292_2 (smooth) on TSV292_1 (rough) using LASTZ alignment tool. Forward alignments between the reference TSV292_1 (rough) contig 1 (**A**) and contig 2 (**B**) (x-axis) and TSV292_2 (smooth) query (y-axis) genomes are shown in blue, indicating regions of conserved synteny in the same orientation. Alignments in red represent sequences aligned in the reverse-complement orientation, corresponding to inverted segments relative to the reference. Gaps with no alignment indicate regions unique to one genome, missing from the other, or below the similarity threshold for alignment.
