# Supplementary material for: Co-isolation of genetically distinct Burkholderia pseudomallei strains from a single patient in North Queensland
Source: PLoS One. 2025 Dec 18;20(12):e0338333. doi: 10.1371/journal.pone.0338333 (PMC12714287; doi:10.1371/journal.pone.0338333)
Supplement: S5 Fig — Highlight of the order of genes with an identified as flagellar product. A) TSV292_2 (smooth) genome. B) TSV292_1 (rough) genome. (PDF) [file pone.0338333.s005.pdf]

A

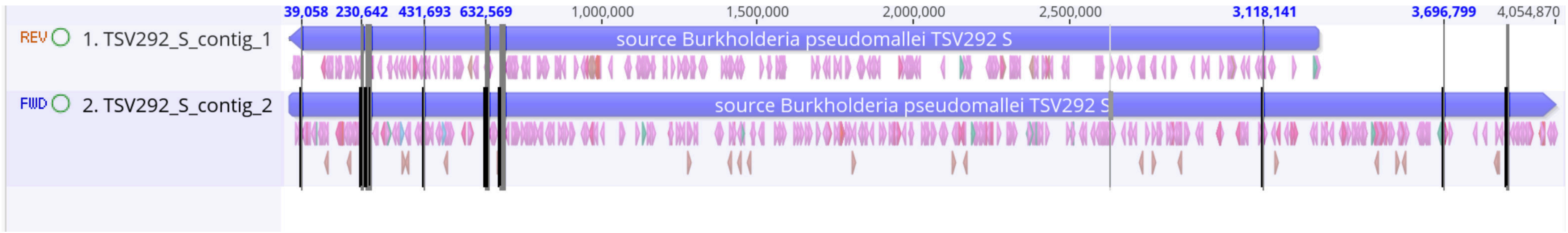

B

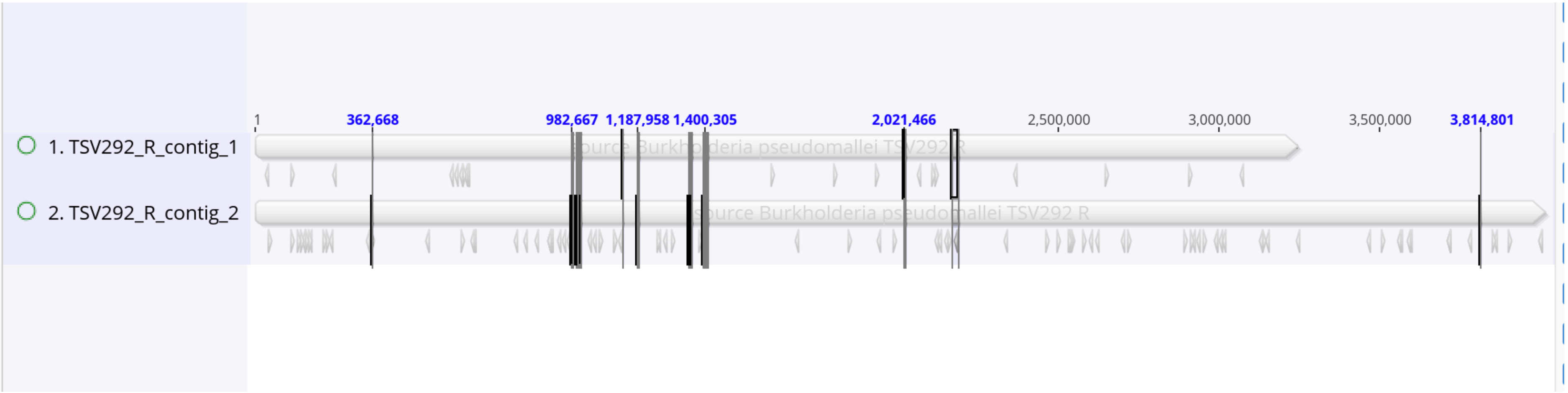

S5 Fig. Highlight of the order of genes with an identified as flagellar product.
A) TSV292_2 (smooth) genome. B) TSV292_1 (rough) genome.
